# Identification of a novel TSC1 variant in a family with developmental and epileptic encephalopathies: A case report and literature review

**DOI:** 10.1097/MD.0000000000040151

**Published:** 2024-10-18

**Authors:** Chao Wang, Jin-Xia Zhai, Yong-Jun Chen

**Affiliations:** a Department of Neurology, The Affiliated Nanhua Hospital, Hengyang Medical School, University of South China, Hengyang, China.

**Keywords:** epilepsy, hamartomas, infantile spasms, TSC1, TSC2, tuberous sclerosis

## Abstract

**Rationale:**

Tuberous sclerosis (TSC) is an autosomal dominant neurocutaneous syndrome resulting from mutations in the tumor suppressor genes *TSC1* and *TSC2*. Unfortunately, the absence of accurate diagnosis has significantly impacted the well-being of both patients and their families. Furthermore, the pathogenicity of numerous variants remains unverified, which could potentially result in misinterpretation of their functional implications.

**Patient concerns:**

Proband 1 was a 33-year-old Chinese male, this patient presents with hamartomas in multiple organ systems, accompanied by clinical symptoms such as intellectual disability, epilepsy, and lipid adenoma. The patient and their family members used targeted next-generation sequencing and Sanger sequencing to identify the pathogenic variant.

**Diagnoses:**

The TSC1 (c.2923G>T, c.2924C>T) variant was identified and the patient was diagnosed with TSC disease.

**Interventions:**

After the definite diagnosis, the patient was treated with valproic acid, oxcarbazepine, and various organ supports.

**Outcomes:**

At present, the patient has intellectual decline, multiple sebaceous adenomas, multiple fiber nodules on the back, palpable mass in the right subcostal and middle upper abdomen, and percussion pain in the right kidney area, 1 to 2 times a month seizure, poor intelligence than peers.

**Lessons:**

This finding strengthens the significant phenotypic variability associated with TSC and expands the mutational spectrum of this rare disease.

## 1. Introduction

Tuberous sclerosis (TSC) is a multisystem genetic disorder characterized by neurological lesions with hamartomas,^[1]^ resulting from mutations in the tuberous sclerosis complex 1 (TSC1, MIM *605284, 9q34.13) or tuberous sclerosis complex 2 (TSC2, MIM *191092, 16p13.3) tumor suppressor genes, often with intellectual disability, epilepsy and autism.^[2,3]^ It was first described in depth in Bernville in 1880, and it is now estimated that nearly 2 million people worldwide are affected by the disease.^[4]^ TSC is inherited in an autosomal dominant fashion, and the clinical features vary widely between individuals, even in the same family. General children often have infantile cramps, white matter abnormalities, cysts, calcification, and subependendymal nodules,^[5]^ most adults suffer from neuropsychiatric disorders and renal angiomyolipoma.^[6]^ With the development of whole-exon sequencing technology, the pathogenic loci of TSC have been detected for many years, but for many people, the overall prognosis is very poor. TSC has been examined for many years with the development of whole-exon sequencing technology, but its ability is limited to screen a large range of the human genome in a single reaction.

Next-generation sequencing (NGS) has made tremendous changes in molecular diagnostics and clinical medicine.^[7]^ It enabled the discovery of novel mutation sites in TSC patients.^[8,9]^ Whole genome sequencing technology focuses on the complete coding regions of the genome, and research meta-analysis plays a key role in the problem of collecting large numbers of samples to discover rare variants associated with complex phenotypes.^[10]^ These technologies have revolutionized areas such as precision medicine, genetic diseases, and clinical diagnostics. However, despite the powerful molecular strategy for identifying genetic mutations, the function of the identified mutations after abnormal mutations in TSC1 or TSC2 in clinically diagnosed TSC patients is not known. This highlights the need for other approaches, such as protein computational modeling and docking analysis, which are able to effectively assess the impact of sequence variation on protein function and help identify sequence variants of unknown biological and clinical significance.

In this study, we report 1 Chinese pedigree of patients with TSC, where targeted NGS and Sanger sequencing revealed a hitherto unreported heterozygous missense variant, TSC1 (c.2923G>T, c.2924C>T). Subsequent computational protein modeling and calmodulin docking analysis confirmed that this abnormal variation led to the generation of abnormal encoded proteins and provided insight into the underlying pathogenic mechanisms of this TSC patient, and this study highlights the importance of genetic testing and alternative molecular analysis techniques in the diagnosis of TSC.

## 2. Patient and methods

### 2.1. Case description

**Figure 1.**
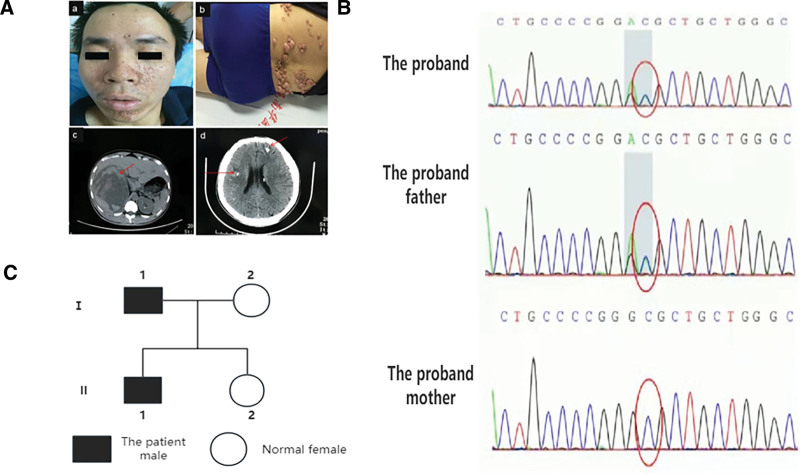
Clinical phenotypes and genetic analysis in the family. (A) Clinical manifestations of the patient with suspected TSC. (B) Family diagram of the patient. (C) Sequencing results of the patient and himself and his father and mother. TSC = tuberous sclerosis.

Proband 1 was a 33-year-old Chinese male who presented to our hospital for treatment because of right-sided abdominal pain with fever. At the age of 3, he often had seizures with a frequency of 3 to 4 times/month, manifested in the form of large seizures, lasting about 1 to 2 minutes. Fifteen to sixteen years took medication at a local hospital, including valproic acid, oxcarbazepine and ketogenic diet, and spontaneously stopped medication after treatment improved. At present, 1 to 2 times a month, poor intelligence than peers, no other special medical history, unmarried and no children. After evaluation in our hospital, physical examination found that the patient had intellectual decline, multiple sebaceous adenomas, multiple fiber nodules on the back, palpable mass in the right subcostal and middle upper abdomen, and percussion pain in the right kidney area. Patient liver and kidney function ALT134U/L, CRP 155 mg/L, urine routine occult blood (+ + +), WBC (+ +), Pro (+ +), electrocardiogram suggests sinus tachycardia, abdominal B ultrasound showed right middle abdomen mixed echo giant mass, abdominal CT suggests renal multiple hamartoma, with right renal giant hamartoma, right liver, pancreatic head compression, deformation, head CT found left frontal lobe and right temporal lobe sheet high density, slightly low density, fifth and sixth ventricle formation, EEG suggests the right central area, apical area, right middle and temporal spine slow wave synchronization, sometimes spread to the left hemisphere. The patient’s parents were not consanguineous, and his father had seizures when he was 3 years of age and had no further seizures after age 7 years, with normal intelligence and normal mother and sister, as shown in Figure 1.

### 2.2. Targeted next-generation sequencing and data analysis

Genomic DNA was extracted from blood samples using the Qiagen DNA Blood Midi/Mini kit (Qiagen GmbH, Hilden, Germany). After rigorous quality control of the DNA samples, sequencing was performed on the Illumina HiSeq X Ten platform (Illumina, San Diego, CA) with an average sequencing depth exceeding 200× and a Q30% > 90%. Sequence reads were aligned to the GRCh37/hg19 reference genome using BWA software. Less reliable variant calls and common variants were filtered out through rigorous bioinformatics analysis. Variant pathogenicity was assessed following the standards and guidelines established by the American College of Medical Genetics and Genomics.

### 2.3. Sanger sequencing

We collected blood samples from the patient and his family and sent them to Beijing Jinzhun Gene Technology Company for whole-exon sequencing, the heterozygous missense mutation of TSC1 gene in proband TSC1 (c.2923G>T, c.2924C>T), reference GenBank sequence (NM_001162427), primers were meticulously designed based on the TSC1 GenBank (NM_001162427) sequence using PRIMER 5 software for PCR amplification. The following variant-specific primers were employed: forward 5′-TAGTGTGCCTGCTCTCTCCT-3′ and reverse 5′-CGGCAGATCACACCTTGAGA-3′. Following purification, the PCR products were subjected to sequencing using the ABI PRISM 3730 Genetic Analyzer.

### 2.4. Computational modeling and docking

The structure of TSC1, the protein encoded by TSC1, was modeled using SWISS-MODEL (https://swissmodel.expasy.org/) to predict the effect of missense variants on protein structure. The model of the ALa975Phe variant was based on the 7DL2pdb template in the Protein Data Bank (PDB) (https://www.rcsb.org/). Hydrogen bonding was analyzed and visualized using PyMOL v.2.3 software (https://pymol.org/2/). After computational modeling, the docking of ALa975Phe with Rheb (PDB: 5yxh) was predicted using GRAMM (http://vakser.compbio.ku.edu/resources/gramm/grammx/). The interaction of the Rheb with wild-type or mutant TSC1 was analyzed. The docking results were visualized using PDBePISA (https://www.ebi.ac.uk/pdbe/pisa/).

## 3. Results

**Figure 2.**
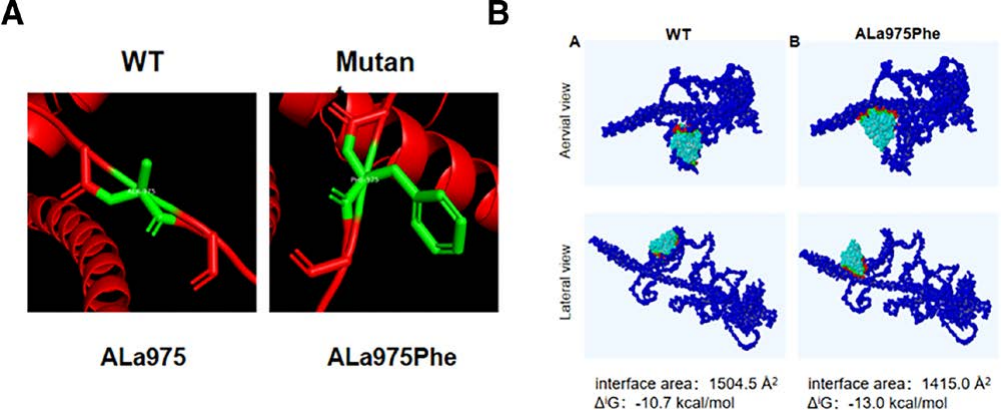
Schematic illustration of hydrogen bonds in TSC1. ALa975 (green) does not form hydrogen bonds with any other residue, and ALa975Phe does not affect hydrogen bonding in TSC1. Model of Rheb docking onTSC1 and the effect of variants. (A) Complex of WT TSC1 and Rheb. (B) Complex of mutant TSC1 and Rheb, ALa975Phe alters the docking site, interface area and Gibbs free energy of TSC1. TSC1 = tuberous sclerosis complex 1, WT = wild-type.

Targeted NGS in the patient revealed a heterozygous candidate variant in *TSC1*: c.2923G>T, c.2924C>T in Exon 25. Sanger sequencing verified this novel heterozygous *TSC1* missense variant, which the patient’s father had the same locus change, and the mother had no corresponding locus change, as shown in Figure 2. Meanwhile this was considered novel because it was not present in the ExAC, 1000G, or HGMD databases.

The TSC complex is the cognate GTP enzyme-activating protein of the small GTP enzyme Rheb,^[11,12]^ and is a key regulator of the target of the rapamycin complex 1 (mTORC1) mechanism. Mutations in the TSC1 and TSC2 subunits of this complex cause TSC.^[13]^ The enriched brain Ras, the homologue (Rheb 1), is a small GTP enzyme that plays a crucial role in regulating cell growth, differentiation, and survival.^[14]^ Alterations in hydrogen bonding caused by ALa975Phe were analyzed using available templates in SWISS-MODEL and PyMOL. TheALa975Phe did not significantly affect hydrogen bonding. The ALa975Phe were located in a core region between TSC1 gene; The ALa975Phe potentially altered the docking site in the Rheb–TSC1 complex, with a significantly alter, decreased interface area (from 1504.5 to 1415.0 Å^2^) but increased Gibbs free energy (Δi*G* from −10.7 to −13 kcal/mol) (Fig. 2).

The correlation between the clinical characteristic of the patient, including multiple hamartomas in both kidneys, ungual fibromas, and a suspected history of epilepsy, with the identified *TSC1* gene variant strongly supports the pathogenicity of the variant in causing the observed phenotype. In line with the American College of Medical Genetics and Genomics guidelines, this variant was unequivocally classified as pathogenic (PVS1 + PM2 + PM6), solidifying its role in the pathogenesis of the patient’s condition.^[15]^

## 4. Discussion

TSC is a disease with widespread manifestations, mainly caused by mutations in genes such as TSC1 and TSC2. TSC1 includes 25 exons and encodes a tuberin protein with a relative molecular weight of 130 kDa.^[16]^ TSC2 includes 42 exons and encodes a hamartin protein with a relative molecular weight of 198 kDa.^[17]^ These 2 proteins bind together to form the TSC complex, involved in the negative regulation of signaling downstream of the mTORC1 receptor.^[18]^ When TSC1 or TSC2 is mutated, the activity of myosin (Rheb) is not inhibited and leads to the phosphorylation of S6K and 4E-BP1,^[19,20]^ which leads to abnormal protein synthesis, loss of control of cell growth, and tumorigenesis.

We report the clinical characteristics and genetic analysis of a TSC patient and his family, with both proband 1 and his father carrying a 2-locus missense mutation TSC1 (c.2923G>T, c.2924C>T). These genetic mutations may affect the structure and function of TSC1 protein, which in turn leads to abnormal activation of mTORC1 signaling, ultimately leading to the development of TSC, and that no somatic mosaicism was observed in other relatives of patient 1, possibly implying that the recombinant relatives may be the father and mother of the proband. However, we cannot exclude the possibility of low levels of somatic mosaicism or germ cell mosaicism in the mothers or grandparents of the probands.^[21]^

NGS technology has significantly improved our ability to identify genetic mutations and variants required for a variety of diseases (including rare and undiagnosed diseases).^[22]^ NGS technology can be used for gene mutation detection, tumor heterogeneity analysis and targeted therapy in cancer, which can help us choose a more suitable treatment plan for patients.^[23,24]^ NGS technology can also be used for genetic detection of genetic diseases, the discovery of new genes and the study of disease etiology, which can help us to better diagnose and treat patients.^[25]^ NGS technology can also be used to study gene expression, proteomics and metabolomics, which can reveal the pathogenesis of the disease and provide potential therapeutic targets and clues for drug development.^[26,27]^ Traditional diagnostic methods may not have the necessary sensitivity to detect these mutations, resulting in an incorrect or incomplete diagnosis.^[28]^ By using NGS technology as a routine diagnostic tool, we can significantly reduce the number of patients without molecular diagnosis and improve their treatment regimen and quality of life.^[29]^

This study analyzed the clinical and genetic characteristics of TSC patients and found that skin lesions and epilepsy were the most typical clinical features of our patients. This is consistent with the findings from previous studies.^[18,30,31]^ Focal seizures, infantile spasticity, and focal to bilateral tonic-clonic seizures are the common types of seizures seen in patients with TSC.^[32]^ One of our patients had a generalized seizure, and he achieved varying degrees of remission after receiving rapamycin treatment.^[33,34]^ One patient presented with multiple hamartomas in both kidneys, resulting in multiple benign tumors in the bilateral kidneys and, besides which, the TSC can affect many other organs, including the lung, liver, retina, as well as the central nervous system.^[35–38]^ The manifestations may include multiple tumors and pneumothorax, and the liver may include multiple tumors and liver cysts. The retina may show things like retinal detachment, macular degeneration, etc.^[39]^ Also, the condition may vary between TSC patients depending on age and genotype.^[40,41]^ Some patients may show more severe cutaneous symptoms, while others may show more severe symptoms of the central nervous system. Two novel mutations were identified in this study.^[42]^ The first mutation, located in the TSC1 gene, occurred at the amino acid at position 975, resulting in alanine to phenylalanine and both bases are altered, namelyTSC1 (c.2923G>T, c.2924C>T). This mutation may disrupt the TSC1 protein expression and function, which in turn leads to the aberrant activation of the mTOR signaling pathway to induce the TSC. The father of proband 1 also carried the mutation. In addition, the use of hydrogen bond docking analysis and the calculation of Gibbs free energy after modeling further verified whether the gene after mutation will affect the function of the protein.^[15]^

## 5. Conclusion

In this study, we identified novel missense mutation sites in TSC1 in a Chinese family using NGS technology, which is important for in-depth analysis of the genetic regulation mechanism of TSC and more accurate diagnosis. Moreover, we also analyzed the clinical characteristics of TSC family and found that the results were consistent with previous studies, suggesting that physicians should pay attention to the typical clinical manifestations of TSC in diagnosis and treatment, especially in cases involving multiple organs. At the same time, we used hydrogen bond analysis and modeling to further verify the pathogenicity of gene mutations, which is expected to provide more useful information for diagnosis and treatment.

In conclusion, our study broadens the genetic spectrum of TSC and provides a more precise basic and therapeutic prospect for the research and treatment of TSC diseases.

## Author contributions

**Conceptualization:** Yong-Jun Chen.

**Data curation:** Chao Wang.

**Visualization:** Jin-Xia Zhai.

**Writing – original draft:** Yong-Jun Chen.
